# Self-reported oral hygiene practice and utilization of dental services by dental technology students in Port Harcourt, Rivers State, Nigeria

**DOI:** 10.4314/ahs.v22i4.33

**Published:** 2022-12

**Authors:** Grace Alade, Sunday Bamigboye

**Affiliations:** University of Port-Hacourt, Preventive Dentistry

**Keywords:** Dental technology, Dental utilization, Oral health practices

## Abstract

**Background:**

Dental technology students are Dental surgery technicians under training, trained to assist patients maintain good oral health. Hence, their oral hygiene is expected to be optimal.

**Objective:**

To determine the oral self-care practices and pattern of utilization of dental services among dental technology students.

**Material and Methods:**

This was a descriptive, cross-sectional study, consisting of 109 participants. The questionnaire had three sections. Section A had questions on sociodemographic, section B was on self-reported oral hygiene practices and section C was on pattern of dental service utilization. Statistical analysis was done using the SPSS version 20.0.

**Results:**

The mean age was 23.66 years; age range was 18 – 42 years. There were 20 males and 89 females, 95.41% of the participants used toothbrushes and paste, while 4.59% used both chewing sticks and toothbrushes. 22% used horizontal/scrub method of toothbrushing, 10.1% used modified bass method, whereas 5.0% do not know which method of toothbrushing they use. 81.3% of the participants had visited the dentist previously for routine dental check-up while 10.71% visited for dental pain.

**Conclusion:**

The self-reported oral hygiene practice of the dental technology students is commendable, however, further education on the importance of scaling and polishing is advocated.

## Introduction

Oral health is an essential part of general health, as, poor oral health has been associated with systemic disease such as cardiovascular disease, diabetes mellitus, etc.^1^ Hence, maintaining good oral hygiene is vital to a sound general health. Oral health is described by the American Dental Association^2^ as being multifaceted and includes the ability to speak, smile, smell, taste, touch, chew, swallow, and convey a range of emotion through facial expressions with confidence and without pain, discomfort, and disease of the craniofacial complex.

The two most common oral diseases of public health importance are dental caries and periodontal diseases, and plaque causes these common oral diseases.^3^ Elimination of plaque is essential in the maintenance of good oral hygiene and hence good oral health. Brushing of teeth twice a day with fluoridated toothpaste and flossing in-between the teeth can help reduce plaque accumulation.^4^ Also, regular dental check-up improves oral health.^5^ Some studies, however, revealed that most individuals utilise dental services when they are in pain or in need of emergency treatment^6^–^8^

Dental auxiliaries are dental personnel that assist the dentist in the management of dental patients. They are classified as non-operating and operating dental auxiliaries.^9^ The operating dental auxiliaries help with professional scaling of teeth (dental therapists), while the non-operating dental auxiliaries (dental surgery technicians) assist the dentists by arranging instruments for dental procedures and giving oral hygiene instructions to patients after dental procedures, hence dental auxiliaries contribute immensely to the care of patients. The dental technology students are dental surgery technicians under training.

A study by Akpata^10^ reported that dental auxiliaries will contribute to the realisation of the oral health vision in Nigeria. They are therefore expected to maintain good oral health behaviour, hence becoming role models for the less educated and the society at large.

There is a paucity in the literature on the oral hygiene practices of the dental technology students. The aim of this study, therefore, is to assess the oral hygiene practice of the dental technology students and to assess their level of utilization of dental services.

## Materials and Methods

This was a descriptive cross-sectional study conducted among dental technology students of Rivers State School of Health Technology, River's state, Nigeria. Ethical approval was obtained from the Health Research and Ethics committee of the University of Port Harcourt Teaching Hospital (UPTH/ADM/90/S. II/VOL.XI/1033), followed by partispant's consent. The study population consisted of 109 dental technology students in the second and final year of the Rivers State School of Health Technology, River's state, Nigeria. The inclusion criteria were students at least 18 years old, dental technology students of Rivers State School of Health Technology and those who gave consent. The investigator administered 110 self-administered questionnaires, but 109 were correctly filled, giving a percentage response of 99%.

The questionnaires were pre-tested among dental students of the University of Port Harcourt, River's state, to ensure simplicity and ease of understanding by the participants. The questionnaire had three sections. Section A had questions on sociodemographic, section B included questions on self-reported oral hygiene practices and section C had questions on the pattern of dental service utilization.

Data analysis was done using Statistical Package for Social Science (SPSS) version 20.0 (IBM SPSS for windows, Armonk, New York, IBM Corp). Descriptive statistics of frequency and percentage were used to present the results.

## Results

The mean age of the subjects was 23.66 years; age range was between 18 – 42 years. There were 20 males and 89 females with M: F of 1:4.45. The mean age for females was 23.27 years, while that for males was 25.40 years, 64.22% of the study population were in 200 level while 35.78% were in 300 level/ final year, 11.93% of the study population are married. This is as shown in table 1.

**Table 1:** Sociodemographic of the participants

Sociodemographic		Frequency (n)	Percentage (%)
**Age group**	≤20	36	33.02
	21–30	67	61.47
	31–40	5	4.59
	>40	1	0.92
**Gender**	Female	89	81.65
	Male	20	18.35
**Level**	200	70	64.22
	300	39	35.78
**Marital status**	Single	96	88.07
	Married	13	11.93
	**Total**	**109**	**100.0**

Almost a ll the participants (95.41%) used toothbrush and paste, none used chewing stick alone, while 4.59% used both chewing stick and toothbrush as cleaning aid, 99.1% brush their teeth twice daily, also 99.1% of the participants used soft/medium textured toothbrush while 0.9% do not know the type of toothbrush being used. Twenty-two percent (22%) of the participants used horizontal/scrub method of toothbrushing, 10.1% used modified bass method, while 59.6% used both horizontal and modified bass methods, whereas 5.0% do not know which method of toothbrushing they use. Majority of the participants (89.9%) changed their toothbrush between 1–3 months, 8.3% change their toothbrush after 3 months, while 1.8% do not know when they change their toothbrush. A large proportion of the participants (97.2%) used dental floss as an interdental cleaning aid, while 2.8% used toothpick as an interdental cleaning aid. (Table 2)

**Table 2:** Oral hygiene maintenance methods of the participants

	Frequency (n)	Percentage (%)
**Cleaning Aids Used**		
Chewing stick Only	0.0	0.0
Toothbrush and paste	104	95.41
Both	5	4.59
Total	109	100.0
**Frequency of brushing**		
Once	1	0.9
Twice	108	99.1
Total	109	100
**Type of toothbrush used**		
Soft/ medium	108	99.1
Hard	0	0.0
Don't know	1	0.9
Total	109	100
**Brushing method used**		
Horizontal/ scrub	24.0	22.0
Modified bass	11	10.1
Both	53	59.6
Don't know	5	5.6
Total	109	100.0
**How often** **toothbrush was changed**		
1–3 Months	98	89.9
>3 Months	9	8.3
Don't know	2	1.8
Total	109	100.0
**Interdental cleaning Aids** **used**		
Dental floss	106	97.2
Toothpick	3	2.8
Total	109	100.0

**Figure 1:**
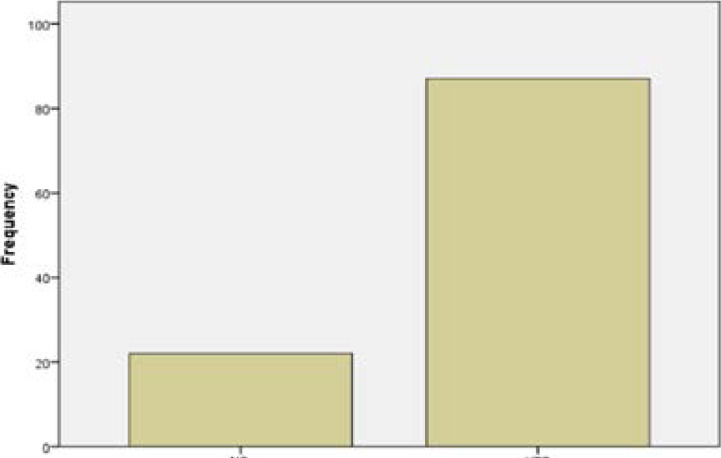
Frequency of scaling and polishing done by participants.

Concerning visitation to the dentist, 93.6% of the participants had visited the dentist previously; majority of this, visited every 6 months and mainly for routine dental check-up (81.37%), while 10.71% visited for dental pain (Table 3), 87% of the participants had scaling and polishing done previously while 23% had not done Scaling and polishing before. (Figure 1)

**Table 3:** Frequency and reasons for dental visit

	Frequency (n)	Percentage (%)
VISIT TO THE DENTIST		
No	7	6.4
Yes	102	93.6
**Total**	**109**	**100.0**
**HOW OFTEN**		
Have Dental Problem	9	8.82
Every six months	78	76.47
Yearly	15	14.71
**Total**	**102**	**100.0**
**REASON FOR LAST** **VISIT**		
Bad breath	1	0.98
Dental pain	11	10.78
Gum bleeding	1	0.98
Hole in my tooth	6	5.88
Routine check up	83	81.37
**Total**	**102**	**100.0**
LAST S&P* DONE		
More than a year ago	11	12.64
Six months to a year ago	11	12.64
Within the last six months	65	74.71
**Total**	**87**	**100.00**

* Scaling and polishing

## Discussion

Oral health is a significant aspect of the general well-being of an individual, as it affects daily activities and productivity such as learning and work. There is an increase in the awareness about oral health recently as global policy has been made to improve oral health.^11^ The dental surgery technicians are important in achieving ideal oral health among dental patients; hence, this study reviews the oral hygiene practice and utilization of dental services of dental technology students, who are dental surgery technicians under training. Also, very few studies have been done among this group.

In this study, majority of the participants cleaned their teeth with toothbrush and paste, this finding is similar to a study carried out among university students, in which all the students clean their teeth with toothbrush and paste, 12 this finding probably reflects their level of education, as the students examined were 200 level and the 300 level (finalists), who were already exposed to sufficient dental education and it will be easier to impart the public with what is already part of their own lifestyle. A few of the participants used both toothbrush and chewing stick to brush, this may be because some people believe that the additional use of chewing stick with toothbrush, produces better result in reducing the amount of food debri.^7^ Chewing stick is known to contain antimicrobial agents that is beneficial for prevention and treatment of periodontal disease.^13^ In addition, a study by Al-Otaibi et al^14^ reported that the use of chewing stick appears to be more effective than tooth brushing for removal of plaque from the embrasures, hence improving interproximal health.

Nearly all the participants brush their teeth twice daily; this result is in agreement with a previous study conducted among dental auxiliaries in Nigeria,^15^ which stated that 71.9% brush their teeth twice daily, but in contrast to other studies conducted among other group of students, in which less than half of the population brushed their teeth twice daily.^12^,^16^ Nearly all the participants use medium toothbrush to clean their teeth, only 10.1% of the population used the modified bass brushing method, however, 6.4% do not know the brushing method they use. This finding is not too encouraging and it may be as a result of less emphasis placed on these participants by their teachers who might have taken it for granted that everyone, including the public from where these students were drawn, ought to have been familiar with the ideal brushing method. Various toothbrushing methods such as fones, horizontal scrub, charters and stillman were reported as effective method of toothbrushing,^17^, while bass or the modified bass technique was reported to be more effective in other studies.^18^,^19^ It has been reported globally, that no particular toothbrushing technique completely removes plaque.^20^ However, there seems to be a consensus agreement that the bass technique cleans the sulcus and subgingival areas.^21^

Majority of the participants change their toothbrushes every three months, this follows the trend of a previous study conducted in Italy,^22^ few change their toothbrush after three months, while others do not know when they ought to change their toothbrush. This finding calls for more emphasis on the importance of changing of toothbrushes after three months, as it has been documented that toothbrushes become frayed after 3 months of usage, as the tooth brushes become old and are less effective. 23 The interdental region is an important area of the oral cavity because it is the area with the highest amount of food and plaque deposits and gingivitis and periodontitis start and occur more in the interdental region of the oral cavity,^24^ it is essential to clean the in-between the teeth as the regular toothbrushes cannot clean this area adequately, Interdental cleaning has been recommended as a supportive aid to toothbrushing to reduce dental plaque accumulation interproximally thereby improving oral health.^25^ In this study, almost all the participants used dental floss to clean in-between their teeth, this is in contrast to previous studies^12^,^16^,^6^,^26^, in which very minute percentage of the participants used dental floss. This difference may be as a result of exposure of the dental technology students to dentistry.

The utilization of dental services is commonly measured by the annual number of dental visits of an individual.^27^ Majority of the participants have visited the dentist before, this is in contrast to previous study among undergraduate students,^12^ in which majority never attended dental clinic. In this present study, most of the participants attended dental clinic within the last six months and for routine dental check-up, this is very commendable and should be encouraged among students, this finding is in contrast to previous studies among dental auxiliaries' students, in which majority attended the dental clinic for scaling and polishing.^28^ Also, in a study by Braimoh et al,^12^ the few undergraduate students who attended dental clinic, did so because of dental pain and extractions. The result from this study could be as a result of the exposure to dentistry. Also, it could be that the routine dental check-up was a requirement for the students in the school.

Majority of the participants had scaling and polishing done in the past. However, there were few who had never done scaling and polishing before, which means that the knowledge of the importance of scaling and polishing is not adequate among dental technology students, who will become dental surgery technicians in future, and are to educate patients on the importance of oral hygiene and preventive dental treatment especially scaling and polishing, which helps in reducing the incidence of preventable oral diseases especially dental caries and periodontal disease. Hence, the dental technology students need to be educated on the importance of scaling and polishing.

## Conclusion

The dental technology students, are the potential dental surgery technicians, which are important in assisting patients maintain good oral health, hence, they are expected to have optimal oral hygiene. The self-reported oral hygiene practices of the dental technology students in this present study are commendable, however, further education on the importance of scaling and polishing is advocated,
